# Impact of perioperative platelet counts and IL-6 on wound healing outcomes after thoracoscopic lung cancer surgery

**DOI:** 10.1080/07853890.2025.2569993

**Published:** 2025-10-31

**Authors:** Bowen Li, Yuanyuan Song, Bin Li, Yu Liu

**Affiliations:** Department of Clinical Laboratory, The Fourth Affiliated Hospital of Harbin Medical University, Harbin, China

**Keywords:** Platelets, inflammatory markers, thoracoscopic surgery, wound healing, lung cancer, perioperative care

## Abstract

**Background:**

Wound healing complications remain significant concerns following thoracoscopic surgery for lung cancer. Perioperative hematological indicators, particularly platelet counts and inflammatory markers, are crucial factors influencing wound healing. Given these gaps, we retrospectively investigated the effect of these indicators on postoperative wound healing outcomes in lung cancer surgery.

**Methods:**

We conducted a retrospective analysis of 200 patients who underwent thoracoscopic surgery for lung cancer. We assessed platelet counts, IL-6, IL-10, TNF-α, and other hematological markers. Patients were stratified by platelet counts into high and low groups. Correlations between these hematological parameters and clinical outcomes, including intraoperative blood loss, hospitalization duration, and wound healing status, were analysed.

**Results:**

Higher perioperative platelet counts were significantly associated with reduced intraoperative bleeding (*p* = 0.015) and shorter hospital stays (*p* = 0.019). The high platelet count group showed better Activities of Daily Living (ADL) scores three days post-surgery (*p* < 0.01). Elevated IL-6 levels preoperatively correlated with adverse wound healing outcomes (*p* < 0.05). Logistic regression analysis indicated that elevated IL-6 was a predictive marker for poor wound healing (OR 1.30, 95% CI 1.15–1.48). Other markers, including IL-10 and TNF-α, were elevated postoperatively in the low platelet count group, indicating a heightened inflammatory response.

**Conclusion:**

Perioperative platelet counts and inflammatory markers, particularly IL-6, significantly influence wound healing in lung cancer surgery. Higher platelet counts were associated with better early postoperative outcomes, while elevated IL-6 levels predict adverse wound healing events. Preoperative IL-6 screening could flag patients who benefit from intensified anti-inflammatory care; multicentre trials with longer follow-up are warranted.

## 1. Introduction

Lung cancer remains one of the leading causes of cancer-related mortality worldwide, with surgical resection being a cornerstone of curative treatment for early-stage disease [1]. Thoracoscopic surgery, often preferred due to its minimally invasive nature, was associated with reduced intraoperative blood loss, shorter hospital stays, and more rapid recovery compared to traditional open thoracotomies [2,3]. However, despite these benefits, postoperative wound healing complications continue to pose significant challenges, impacting patient recovery and long-term outcomes [4]. Common complications include dehiscence, surgical site infection and delayed epithelialisation; excessive IL-6 driven inflammation exacerbates these events by impairing angiogenesis [5].

Wound healing was a complex and dynamic process involving hemostasis, inflammation, proliferation, and remodeling [6]. Integral to this process were various cellular and molecular mechanisms, many of which were influenced by perioperative hematological factors [7]. Platelets, as key players in hemostasis, initiate clot formation and release growth factors that recruit cells essential for tissue repair [8]. Furthermore, platelets modulate the inflammatory response, which was crucial for clearing debris and preventing infection but, if dysregulated, can impede healing through prolonged or excessive inflammation [9,10].

Recent studies have highlighted the role of systemic inflammatory responses in postoperative outcomes [11]. Inflammatory markers such as interleukin-6 (IL-6), interleukin-10 (IL-10), and tumour necrosis factor-alpha (TNF-α) have been associated with a variety of surgical complications, including impaired wound healing [12,13]. Elevated levels of these cytokines can signal an exaggerated inflammatory response, leading to tissue damage, fibrosis, and delayed wound closure [14].

In the context of lung cancer surgery, understanding the relationship between perioperative hematological indicators and wound healing was particularly pertinent [15]. Hematological parameters, including platelet counts, white blood cell (WBC) counts, and cytokine levels, fluctuate during the perioperative period, influenced by surgical trauma, anaesthesia, and the body’s intrinsic response to injury [16]. Identifying specific hematological factors that predict or correlate with wound healing outcomes could facilitate early intervention and individualized patient management, potentially improving surgical results and patient quality of life [17,18].

Despite the recognized importance of hematological factors in wound healing, research specifically addressing their role in lung cancer surgery remains relatively sparse [19]. Most existing studies focus on broader surgical populations or do not adequately differentiate between the effects of various hematological indicators [20]. Given the unique physiological and immunological challenges posed by lung cancer and thoracoscopic surgery, a dedicated investigation into these factors’ impact on wound healing was warranted.

Our study aimed to bridge this gap by examining the effect of perioperative hematological indicators on postoperative wound healing in patients undergoing thoracoscopic surgery for lung cancer. We hypothesize that specific hematological parameters, particularly those related to platelet function and systemic inflammation, significantly influence wound healing outcomes.

## 2. Materials and methods

### 2.1. Case selection

This retrospective cohort study included 200 patients who underwent thoracoscopic surgery for lung cancer at our hospital from January 2020 to January 2022. Demographic data, pre-surgery pulmonary function, pre-surgery blood routine indices, perioperative inflammatory response indicators, observation indices, wound healing in two post-surgery patient groups, and Activities of Daily Living (ADL) scores were systematically collected. Since this retrospective study only involved de-identified patient data, with no potential harm or impact on patient care, informed consent was waived. Both the waiver and the study were approved by the hospital’s Ethics Review Committee and Ethics Committee, adhering to regulatory and ethical guidelines for retrospective research.

#### 2.1.1. Ethics statement

All research is in strict accordance with and adheres to the Declaration of Helsinki.

The study was approved by the Ethics Committee of The Fourth Affiliated Hospital of Harbin Medical University(HLJ-HB-2024-022).

### 2.2. Inclusion and exclusion criteria

Inclusion criteria consisted of participants aged between 20 and 70 years old, without a history of mental illness, possessing normal cognitive abilities, able to cooperate with treatments and examinations. Patients classified under American Society of Anaesthesiologists (ASA) Physical Condition (PS) Class I or II were considered. Diagnosis was confirmed either through cytology or clinical pathology, accompanied by typical clinical manifestations and lung cancer symptoms. Patients had not undergone prior chest surgery and were undergoing thoracoscopic radical surgery for lung cancer for the first time, without preceding radiotherapy or chemotherapy. Tumour lesion diameter was less than 5 cm with no distant metastasis, and participants willingly agreed to partake in the study.

Exclusion criteria entailed individuals displaying unstable vital signs like heart rate, body temperature, and blood pressure. Those with severe cognitive impairment, a history of mental illness, or extended use of certain medications were excluded. Patients exhibiting coagulation dysfunction or severe liver, kidney, or heart issues were not included. Additionally, those with conditions such as pulmonary diseases, chest adhesions, pleural thickening, or calcification, as well as those with specific cardiac conditions or allergies to anaesthesia drugs were excluded from the study.

The patient selection and follow-up process is summarized in Supplementary Figure S1.

Supplementary Figure S1 The patient selection and follow-up process

### 2.3. Grouping criteria

Based on pre-surgery blood routine test results, patients were categorized into two groups: the high platelet count group comprising 106 individuals and the low platelet count group comprising 94 individuals. The high platelet count group had platelet counts ranging from 135 to 235 × 10^9/L, while the low platelet count group had platelet counts ranging from 236 to 335 × 10^9/L. These cut-offs correspond to the inter-quartile boundaries in our pre-study audit and align with prior thoracic surgery norms, where platelet levels are typically stratified based on clinical thresholds or IQR boundaries [21,22]. A sensitivity analysis using tertile grouping showed similar results (Supplementary Table S1).

### 2.4. Pulmonary function testing

A full lung function analyser from Jaeger, Inc. in Bodnegg, Germany was utilized to assess different lung function parameters. The study evaluated the lung function metrics of two patient cohorts before and after treatment, encompassing FEV1, FVC, FEV1/FVC ratio, DLCO, among others.

### 2.5. Anaesthesia methods

All patients adhered to an 8-hour fasting period and consumed liquids up to 4 h before the surgical procedure. Upon admission to the operating room, venous access was established, and a single-port thoracoscopic surgery for lung cancer was conducted, involving an incision on the anterior chest wall. General anaesthesia induction comprised administering 1 mg/kg rocuronium bromide, 2 mg/kg/min propofol, 0.5 μg/kg sufentanil, and 0.05 mg/kg midazolam intravenously. Anaesthesia maintenance was achieved through a targeted infusion of remifentanil (with a target concentration of 2–3 ng/ml in the effect chamber) and propofol (target concentration of 2–3 μg/ml in plasma), supplemented intermittently with sufentanil and atracurium. Subsequent to the surgery, intravenous patient-controlled analgesia was facilitated for 48 h, with a single 2 ml dose and a lockout period of 15 min. The basal rate stood at 2 ml/h, a 2 ml loading dose was administered, and a maximum of 8 ml/h was ensured. The postoperative analgesic regimen involved 100 ml of physiological saline, 5 mg of tropisetron, and 1 μg/kg of sufentanil.

### 2.6. Thoracoscopic wedge resection of lung

Prior to surgery, a panel of three experienced thoracic surgeons evaluated the necessity of nodule localization using CT guidance and Hook-Wire. Subsequently, the patient was swiftly transferred to the operating room for the surgical procedure. Employing dual lumen tracheal intubation, the patient was positioned laterally in a medically sound manner while under a combination of intravenous and inhalation general anaesthesia. The surgeries were conducted by physicians belonging to the same surgical team utilizing a sole thoracoscopy approach in the fourth intercostal space along the anterior axillary line. During the procedure, a wedge-shaped excision of the tumour, positioned 2.0 cm away from its margins, was carried out based on the guide wire’s exploration and placement. Post-cavity irrigation, a 12 Fr pigtail tube was inserted between the 7th rib at the midaxillary line, followed by layered chest closure.

### 2.7. Blood testing

In the study, blood samples were collected from patients on the day before surgery, intraoperatively, as well as one and two days post-surgery, after an overnight fast. The samples, each consisting of 5 ml of venous blood, underwent various analyses. Red blood cell, Neutrophil, Lymphocyte, Eosinophil, Basophil, Haemoglobin, Platelet, and WBC levels were measured using the DxH800 Haematology Analyser (Beckman Coulter, Inc., Brea, CA, USA). Whole blood samples anticoagulated with ethylenediaminetetraacetic acid (EDTA) were utilized to determine the Erythrocyte Sedimentation Rate (ESR) using the TEST1 automated sedimentation rate analyser (ALIFAX, Inc., Italy). Plasma samples were obtained by centrifugation at 3,000 rpm for 5 min, and the supernatant was used for the detection of TNF-α, IL-6, and IL-10. Enzyme-linked immunosorbent assay (ELISA) kits for TNF-α (ab181421, abcam, USA), IL-6 (ab178013, abcam, USA), and IL-10 (ab185986, abcam, USA) were employed for this purpose.

### 2.8. ADL score

Measurement of ADL score involved utilizing a scale to assess the patient’s daily activity capacity through six items. Each item was rated on a scale where complete independence scored 1 point, dependence on assistance was scored as 0 points, leading to a maximum total score of 6 points. A higher score indicated a greater functional ability. The reliability of the ADL score was reported to be exceptionally high at 0.99 [23].

### 2.9. Statistical method

The data measurements were presented as mean ± standard deviation or median interquartile range, depending on their distribution. Categorized data was shown in frequency and percentage. Comparison between two groups for continuous variables was done using unpaired t-tests. Statistical analyses involved univariate and multivariate logistic regression to determine the odds ratio (OR) and 95% confidence interval (CI) for each continuous parameter. Logistic regression was used to model the relationship between IL-6 levels and wound healing outcomes, adjusting for tumour stage, histology, age, and sex to isolate the IL-6 effect. Statistical significance was set at *p* < 0.05. The analyses were carried out using SPSS 19 software (SPSS Inc., Chicago, IL, USA) and R software package 3.0.2 (Free Software Foundation, Inc, Boston, MA, USA).

### 2.10. Wound healing evaluation

Wound healing was assessed using the Southampton wound scoring system (grades 0–5), where grade 0 indicates normal healing, grade I indicates mild bruising or erythema, grade II indicates other signs of inflammation, grade III indicates clear or haemoserous discharge, grade IV indicates pus, and grade V indicates deep or severe wound infection [24]. Poor wound healing was defined as Southampton score ≥ II at postoperative day 3.

## 3. Results

### 3.1. Analysis of general data

In comparing the general information between the high platelet count group (*n* = 106) and the low platelet count group (*n* = 94) in the study on the effect of perioperative hematological indicators on wound healing in lung cancer surgery, there were no statistically significant differences observed in terms of baseline demographic and clinical characteristics, as detailed in Table 1. No recurrence data were available within the 90-day follow-up period, which is acknowledged as a limitation of this study.

**Table 1.** Comparison of general information between two groups.

Parameter	High platelet count (*n* = 106)	Low platelet count (*n* = 94)	T	*p*
Age (years)	45.47 ± 19.52	45.50 ± 19.48	0.255	0.799
BMI (kg/m²)	24.73 ± 3.30	24.55 ± 3.21	0.664	0.507
Education Level (years)	13.36 ± 3.62	13.33 ± 3.54	0.103	0.918
Gender			0.052	0.82
- Male	57(53.77%)	53(56.38%)		
- Female	49(46.23%)	41(43.62%)		
Hypertension			0.226	0.635
- Yes	35(33.02%)	35(37.23%)		
- No	71(66.98%)	59(62.77%)		
Valvular Heart Disease			0.186	0.666
- Yes	32(30.19%)	32(34.04%)		
- No	74(69.81%)	62(65.96%)		
Diabetes Mellitus			0.07	0.792
- Yes	21(19.81%)	21(22.34%)		
- No	85(80.19%)	73(77.66%)		
Smoking history	67(63.21%)	57(60.64%)	0.052	0.82
Drinking history	42(39.62%)	41(43.62%)	0.184	0.668
6-minute walk distance (m)	424.3 ± 30.74	423.68 ± 30.76	0.143	0.887
Lesion size(mm)	10.80 ± 3.30	10.70 ± 2.60	0.244	0.808
Tumour Stage			0.312	0.856
- Stage I	58(54.72%)	49(52.13%)		
- Stage II	35(33.02%)	33(35.11%)		
- Stage III	13(12.26%)	12(12.76%)		
Histology			0.184	0.668
- Adenocarcinoma	72(67.92%)	61(64.89%)		
- Squamous cell carcinoma	34(32.08%)	33(35.11%)		

### 3.2. Lung function on the day before surgery

Comparing lung function between the high platelet count group and the low platelet count group in the study on the effect of perioperative hematological indicators on wound healing in lung cancer surgery, no statistically significant differences were found in forced expiratory volume in one second (FEV1) (0.84 ± 0.11 vs. 0.85 ± 0.10, 1.151, *p* = 0.251), forced vital capacity (FVC) (1.57 ± 0.21 vs. 1.55 ± 0.20, 0.526, *p* = 0.6), FEV1/FVC ratio (48.19 ± 6.07 vs. 48.21 ± 6.13, 0.026, *p* = 0.979), and diffusing capacity of the lungs for carbon monoxide (DLCO) (15.85 ± 1.58 vs. 15.99 ± 1.55, 0.638, *p* = 0.524) on the day before surgery. These results indicate that preoperative lung function parameters were comparable between the high and low platelet count groups in the cohort (Supplementary Table S2).

### 3.3. Blood routine examination indicators on the first day before surgery

In the blood routine examination indicators comparison between the high platelet count group and the low platelet count group on the first day before surgery in the investigation of the effect of perioperative hematological indicators on wound healing in lung cancer surgery, significant differences were observed in platelet levels (176.82 ± 42.74 vs. 267.63 ± 43.13, 14.924, *p* < 0.001). However, there were no statistically significant variances in erythrocyte sedimentation rate (ESR) (35.83 ± 5.36 vs. 34.76 ± 4.98, 1.472, *p* = 0.143), red blood cell count (5.44 ± 1.59 vs. 5.32 ± 1.67, 0.527, *p* = 0.599), neutrophil count (4.32 ± 1.06 vs. 4.37 ± 1.08, 0.35, *p* = 0.726), lymphocyte count (2.03 ± 0.68 vs. 2.09 ± 0.71, 0.665, *p* = 0.507), eosinophil count (0.28 ± 0.03 vs. 0.28 ± 0.03, 0.443, *p* = 0.658), basophil count (0.09 ± 0.03 vs. 0.09 ± 0.03, 0.402, *p* = 0.688), and haemoglobin levels (149.41 ± 24.84 vs. 149.85 ± 25.37, 0.124, *p* = 0.901) between the two groups (Supplementary Table S3).

### 3.4. Intraoperative and postoperative observation indicators

In comparing intraoperative and postoperative observation indicators between the high platelet count group and the low platelet count group in patients undergoing thoracoscopic wedge resection of the lung in the analysis of the impact of perioperative hematological indicators on wound healing in lung cancer surgery, significant differences were noted in intraoperative bleeding volume (26.72 ± 12.76 vs. 31.35 ± 13.81, 2.451, *p* = 0.015) and hospitalization time (4.56 ± 0.80 vs. 4.85 ± 0.92, 2.374, *p* = 0.019) between the two groups (Table 2). However, there were no statistically significant discrepancies observed in surgical time (61.12 ± 12.35 vs. 62.48 ± 13.79, 0.729, *p* = 0.467), thoracic drainage volume (164.38 ± 73.58 vs. 148.71 ± 62.28, 1.631, *p* = 0.104), placement time of thoracic drainage tube (2.59 ± 0.64 vs. 2.49 ± 0.75, 0.945, *p* = 0.346), or incidence of lower limb venous thrombosis (3.77% vs. 3.19%, 0, *p* = 1) between the groups.

**Table 2.** Comparison of intraoperative and postoperative observation indicators between two groups of patients undergoing thoracoscopic wedge resection of the lung.

Parameter	High platelet count (*n* = 106)	Low platelet count (*n* = 94)	T	*p*
Intraoperative bleeding volume (ml)	26.72 ± 12.76	31.35 ± 13.81	2.451	0.015
Surgical time (min)	61.12 ± 12.35	62.48 ± 13.79	0.729	0.467
Thoracic drainage volume (ml)	164.38 ± 73.58	148.71 ± 62.28	1.631	0.104
Placement time of thoracic drainage tube (d)	2.59 ± 0.64	2.49 ± 0.75	0.945	0.346
Hospitalization time (d)	4.56 ± 0.80	4.85 ± 0.92	2.374	0.019
Lower limb venous thrombosis [*n* (%)]	4(3.77%)	3(3.19%)	0	1

### 3.5. Comparison of inflammatory response indexes in each period of operation

**Figure 1.**
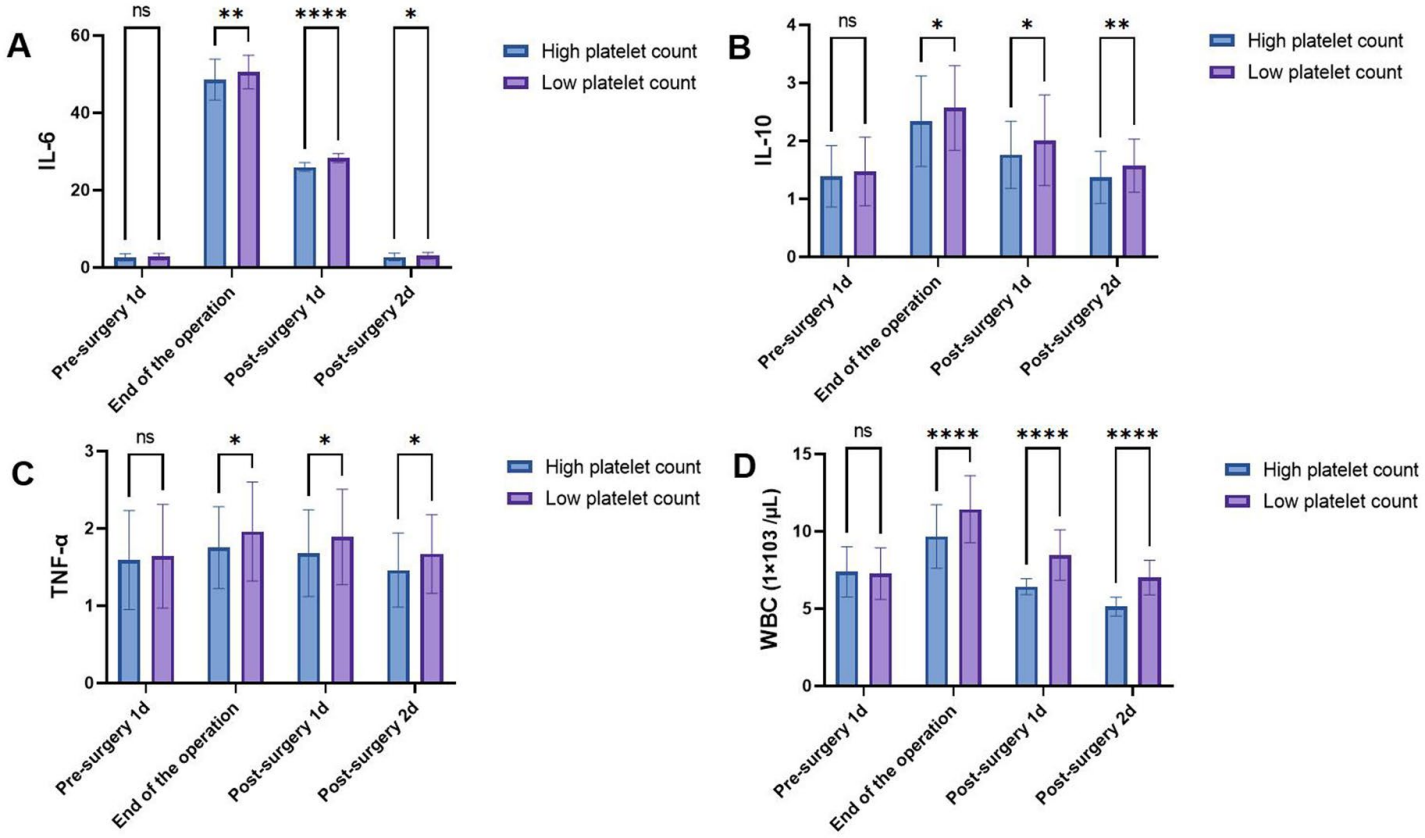
Comparison of inflammatory markers between high and low platelet count groups across perioperative periods. A) IL-6 levels, B) IL-10 levels, C) TNF-α levels, D) WBC counts. Error bars represent standard deviation. *p < 0.05, **p < 0.01, ***p < 0.001.

In evaluating inflammatory response indicators between patients with high platelet count and low platelet count undergoing lung cancer surgery, on the day before surgery, no significant differences were found in IL-6 (2.65 ± 0.93 vs. 2.79 ± 0.82, 1.142, *p* = 0.255), IL-10 (1.39 ± 0.53 vs. 1.47 ± 0.59, 0.907, *p* = 0.366), TNF-α (1.59 ± 0.64 vs. 1.64 ± 0.67, 0.572, *p* = 0.568), and WBC (7.38 ± 1.62 vs. 7.26 ± 1.67, 0.525, *p* = 0.6). However, at the end of surgery, significant differences were observed in IL-6 (48.62 ± 5.29 vs. 50.56 ± 4.32, 2.851, *p* = 0.005), IL-10 (2.34 ± 0.78 vs. 2.57 ± 0.73, 2.179, *p* = 0.03), TNF-α (1.75 ± 0.53 vs. 1.96 ± 0.64, 2.577, *p* = 0.011), and WBC (9.67 ± 2.05 vs. 11.43 ± 2.16, 5.875, *p* < 0.001), showing higher levels in patients with low platelet count. Similarly, on the first and second days after surgery, significant differences were noted in IL-6, IL-10, TNF-α, and WBC, with higher values in the low platelet count group compared to the high platelet count group across all time points. These results suggest a potential relationship between platelet count and inflammatory response dynamics following lung cancer surgery (Figure 1).

### 3.6. ADL scores before and after treatment

In examining ADL scores between patients with high platelet count and low platelet count undergoing lung cancer surgery, no significant differences were detected in ADL scores on the day before surgery (3.90 ± 1.23 vs. 3.89 ± 1.12, 0.072, *p* = 0.943). However, postoperatively at day 3, a significant disparity was observed in ADL scores (5.42 ± 1.25 vs. 5.01 ± 1.24, 2.324, *p* = 0.021), indicating better ADL performance in the high platelet count group (Table 3). Conversely, by day 7 post-surgery, no statistically significant variances were noted in ADL scores (5.65 ± 0.95 vs. 5.61 ± 0.78, 0.307, *p* = 0.759). These findings suggest a transient improvement in ADL scores in patients with high platelet count shortly after lung cancer surgery, emphasizing the potential role of platelet count in the early recovery phase.

**Table 3.** Comparison of ADL scores between two groups of patients before and after treatment.

Parameter	High platelet count (*n* = 106)	Low platelet count (*n* = 94)	T	*p*
Pre-surgery1d	3.90 ± 1.23	3.89 ± 1.12	0.072	0.943
Post-surgery3d	5.42 ± 1.25	5.01 ± 1.24	2.324	0.021
Post-surgery7d	5.65 ± 0.95	5.61 ± 0.78	0.307	0.759

### 3.7. Comparison of poor wound healing between the two groups at post-surgery 3d

Comparing poor wound healing outcomes between patients with high platelet count and low platelet count three days postoperatively, there were no statistically significant differences observed in local exudation, pain, fever, wound dehiscence, or overall adverse event rate, which was 6.60% in the high platelet count group and 8.51% in the low platelet count group (*T* = 0.059, *p* = 0.809) (Table 4). These results indicate no significant association between platelet count and poor wound healing within the early postoperative period after lung cancer surgery.

**Table 4.** Comparison of poor wound healing between two groups of patients in postoperative 3 days.

Group	Local exudation	Pain	Fever	Wound dehiscence	Overall adverse event rate [n/%]
High platelet count (n = 106)	3	2	1	1	7(6.60%)
Low platelet count (n = 94)	2	3	2	1	8(8.51%)
T					0.059
P					0.809

### 3.8. Influence of perioperative hematological indexes on wound healing in lung cancer surgery

In assessing the impact of perioperative hematological indicators on wound healing in lung cancer surgery, no statistically significant differences were observed in ESR, red blood cell count, neutrophil count, lymphocyte count, eosinophil count, basophil count, haemoglobin levels, and platelet count between patients with normal wound healing and those with adverse wound healing (Table 5). However, a significant difference was noted in the IL-6 levels at baseline (2.68 ± 0.87 vs. 3.19 ± 0.92, *t* = 2.175, *p* = 0.031), whereas no significant differences were found in IL-10, TNF-α, and WBC levels preoperatively or at the end of surgery.

**Table 5.** Influence of perioperative hematological indexes on wound healing in lung cancer surgery.

Parameter	Normal wound healing	Adverse wound healing	t	*p*-value
ESR (mm/h)	35.36 ± 5.05	34.89 ± 7.05	0.257	0.801
Red blood cell (1 × 10⁶ /μL)	5.4 ± 1.64	5.19 ± 1.53	0.466	0.642
Neutrophil (1 × 10³ /μL)	4.36 ± 1.08	4.22 ± 0.93	0.489	0.625
Lymphocyte (1 × 10³ /μL)	2.06 ± 0.69	2.04 ± 0.69	0.126	0.9
Eosinophil(1 × 10² /μL)	0.28 ± 0.03	0.29 ± 0.03	1.353	0.178
Basophil (1 × 10/μL)	0.09 ± 0.03	0.1 ± 0.03	1165	0.301
Haemoglobin (g/L)	150.13 ± 25.35	143.35 ± 20.32	1.009	0.314
Platelet (1 × 10³ /μL)	219.8 ± 60.41	215.76 ± 86.2	0.178	0.861
Pre-surgery 1d IL-6	2.68 ± 0.87	3.19 ± 0.92	2.175	0.031
Pre-surgery 1d IL-10	1.43 ± 0.56	1.35 ± 0.54	0.553	0.581
Pre-surgery 1d TNF-α	1.61 ± 0.65	1.7 ± 0.76	0.528	0.598
Pre-surgery 1d WBC(1 × 10³ /μL)	7.35 ± 1.65	7 ± 1.47	0.792	0.43
End of the operation IL-6	49.62 ± 4.86	48.5 ± 6.01	1475	0.687
End of the operation IL-10	2.45 ± 0.75	2.36 ± 0.91	0.468	0.64
End of the operation TNF-α	1.85 ± 0.59	1.84 ± 0.72	0.055	0.956
End of the operation WBC(1 × 10³ /μL)	10.47 ± 2.22	10.88 ± 2.94	0.681	0.497

In logistic regression analysis (Table 6), a significant association was found between preoperative 1-day IL-6 levels and adverse wound healing events, with a coefficient of 0.684 (standard error 0.322, Wald = 2.122, *p* = 0.034) and an odds ratio of 1.981 (95% CI 1.071–3.825), indicating a potential predictive role of preoperative 1-day IL-6 in wound healing outcomes following lung cancer surgery. After adjusting for tumour stage, histology, age, and sex as covariates, the main IL-6 finding remained unchanged (see Supplementary Table S4 for full model summary).

**Table 6.** Logistic regression of preoperative 1d IL-6 and adverse wound healing event.

Parameter	Pre-surgery 1d IL-6
Coefficient	0.684
Std Error	0.322
Wald	2.122
P Value	0.034
OR (95% CI)	1.981 (1.071–3.825)

## 4. Discussion

The effect of perioperative hematological indicators on wound healing in lung cancer surgery was a topic of significant clinical relevance [25]. Our study retrospectively examined patients who underwent thoracoscopic surgery for lung cancer, with particular attention paid to a range of demographic, clinical, and perioperative hematological factors. One of our primary discoveries was the differential impact of platelet counts and systemic inflammatory responses on wound healing and recovery, which provides valuable insights into the complex interactions between hematological parameters and surgical outcomes.

The differentiation of patients into high and low platelet count groups enabled us to dissect the influence of platelet levels on wound healing. Platelets play an essential role in hemostasis and were integral to the wound healing process through their involvement in clot formation, inflammatory response modulation, and tissue regeneration [26,27]. Higher platelet counts were associated with reduced intraoperative bleeding and shortened hospitalization times. This can be attributed to the fundamental role of platelets in clot formation, which diminishes bleeding time and facilitates faster initial wound stabilization [28,29]. Additionally, the presence of sufficient platelets aids in the more efficient recruitment of essential molecules and cells to the wound site, which accelerates the healing process [30].

The mechanistic basis for platelet-mediated wound healing involves multiple pathways. Platelets serve as one of the most abundant sources of TGF-β among all normal tissues [31]. During surgical hemostasis, platelet α-granules immediately release TGF-β, establishing the foundation for subsequent fibroblast and exosome-mediated matrix deposition [32]. Additionally, myofibroblasts actively produce inflammation-supporting factors, notably IL-6, thereby shaping a microenvironment conducive to inflammation [5]. The IL-6/STAT3 pathway promotes keratinocyte proliferation and angiogenesis; our finding of elevated IL-6 in patients with poor wound healing corroborates its potential inhibitory role in lung incision healing [33].

However, our results also indicated that elevated platelet counts were linked to better ADL scores three days post-surgery, implying a more rapid return to functional independence. This transient improvement, however, did not persist beyond the early postoperative period, as evidenced by the ADL scores observed at seven days post-surgery, which showed no significant differences between the groups. The initial postoperative phase was critical for functions like mobilization and pain management, where platelet-mediated clot stability can minimize complications such as haemorrhage and infection, contributing to a quicker early functional recovery [34,35].

The inflammatory response was another crucial factor influencing wound healing [36,37]. This study utilized several key inflammatory markers, including IL-6, IL-10, TNF-α, and WBC, to monitor perioperative inflammatory status. We observed that the low platelet count group exhibited significantly higher levels of these inflammatory markers at various time points during and after surgery. Increased levels of these cytokines and WBCs suggest a heightened systemic inflammatory response, which could potentially impede wound healing through several mechanisms.

IL-6, in particular, emerged as a pivotal marker, with elevated preoperative levels correlating strongly with adverse wound healing outcomes [38]. IL-6 was known for its dual role in inflammation, acting both as a pro-inflammatory cytokine and an anti-inflammatory mediator depending on the context [39]. The augmented levels of IL-6 in the low platelet count group suggest that these patients might have undergone excessive or prolonged inflammation, which can lead to impaired tissue repair and increased postoperative complications [40,41]. Such an exaggerated inflammatory response can result in excessive tissue damage, fibrosis, and delayed re-epithelialization—key factors that compromise effective wound healing [42,43].

Furthermore, the logistic regression analysis confirmed the potential of IL-6 as a predictive marker for adverse wound healing events. This lays the foundation for the utilization of preoperative IL-6 levels in risk stratification and pre-emptive therapeutic interventions. Targeted anti-inflammatory strategies could be tailored for patients identified with high preoperative IL-6 levels to mitigate the risk of poor wound healing.

The post-surgical elevation in inflammatory markers in the low platelet count group underlines the intricate interplay between hemostasis and inflammation. Platelets release bioactive molecules, including growth factors and cytokines like Platelet-Derived Growth Factor (PDGF) and Transforming Growth Factor-beta (TGF-β), which modulate the inflammatory milieu and foster tissue repair [44,45]. A deficiency in platelets may hinder the adequate release of these mediators, thus contributing to an unregulated inflammatory response [46].

The role of IL-10 also demands attention. As an anti-inflammatory cytokine, IL-10 functions to restrain excessive immune responses and limit tissue damage [47]. The increased levels of IL-10 observed postoperatively in the low platelet count group might represent a compensatory mechanism attempting to counterbalance the heightened inflammation [48]. However, this compensation may not be sufficient to offset the primary inflammatory drive, leading to suboptimal healing outcomes.

TNF-α, a master regulator of inflammation, was also found to be elevated post-surgery among patients with lower platelet counts [49]. TNF-α exerts its effects by promoting leukocyte recruitment, inducing the production of other cytokines, and orchestrating the overall inflammatory response [50]. Elevated TNF-α levels indicate a pro-inflammatory state that, if uncontrolled, can result in chronic inflammation and impaired wound healing due to persistent tissue damage and inhibition of the regenerative phase [51].

The findings of this study underscore the importance of perioperative hematological monitoring, particularly platelet and cytokine levels, as potential prognostic tools for wound healing outcomes in lung cancer surgery. Clinicians should consider incorporating routine preoperative assessments of inflammatory markers like IL-6 alongside traditional hematological tests to identify high-risk patients. Pre-emptive measures, including anti-inflammatory therapies and personalized postoperative care protocols, can then be designed to improve healing outcomes.

Future research should further explore the mechanistic pathways linking platelet function and inflammatory responses to wound healing. Investigating the interplay of platelets and cytokines on a cellular and molecular level provide deeper insights into potential therapeutic targets. Additionally, clinical trials evaluating anti-inflammatory interventions in patients with high preoperative IL-6 levels could elucidate effective strategies to enhance postoperative recovery and wound healing.

While the findings of our study provide valuable insights into the impact of perioperative hematological indicators on wound healing in lung cancer surgery, several limitations should be acknowledged. Firstly, the retrospective nature of our study may introduce selection bias and limit the generalisability of our results. Secondly, the study was conducted at a single medical centre, which may constrain the applicability of our findings across different populations and healthcare settings. Additionally, our analysis relied on standard hematological and inflammatory markers without considering other potential biological or genetic factors that could influence wound healing. The relatively short follow-up period may also have prevented the observation of long-term wound healing outcomes and complications. The absence of ≥12 month follow-up data limits our ability to assess the durability of wound healing outcomes. Future prospective validation studies with extended follow-up periods are planned to address this limitation.

## 5. Conclusion

In conclusion, our study highlights the pivotal roles of platelet counts and inflammatory responses in influencing wound healing in lung cancer surgery. By understanding these mechanisms, we can better predict and manage postoperative outcomes, ultimately improving patient care and recovery in thoracic surgical operations. Preoperative IL-6 screening could flag patients who benefit from intensified anti-inflammatory care. Multicentre trials with longer follow-up are warranted to validate these findings and establish standardized protocols for perioperative management.

## Supplementary Material

Supplemental Material

Supplemental Material

## Data Availability

The datasets used and/or analysed during the current study are available from the corresponding author on reasonable request.
