# Supplementary material for: Impact of perioperative platelet counts and IL-6 on wound healing outcomes after thoracoscopic lung cancer surgery
Source: Ann Med. 2025 Oct 31;57(1):2569993. doi: 10.1080/07853890.2025.2569993 (PMC12581744; doi:10.1080/07853890.2025.2569993)
Supplement: Supplemental Material [file IANN_A_2569993_SM1624.docx]

**Supplementary Table S1. Comparison of lung function between two groups of patients on the day before surgery**

| Parameter | High platelet count (n=106) | Low platelet count (n=94) | T | P |
| --- | --- | --- | --- | --- |
| FEV1(L) | 0.84 ± 0.11 | 0.85 ± 0.10 | 1.151 | 0.251 |
| FVC(L) | 1.57 ± 0.21 | 1.55 ± 0.20 | 0.526 | 0.600 |
| FEV1/FVC(%) | 48.19 ± 6.07 | 48.21 ± 6.13 | 0.026 | 0.979 |
| DLCO[ml/(min·mm Hg)] | 15.85±1.58 | 15.99±1.55 | 0.638 | 0.524 |

Sensitivity analysis with tertile grouping:

| Parameter | Low tertile (n=67) | Middle tertile (n=66) | High tertile (n=67) | F | P |
| --- | --- | --- | --- | --- | --- |
| FEV1(L) | 0.83 ± 0.12 | 0.85 ± 0.10 | 0.85 ± 0.11 | 0.642 | 0.527 |
| FVC(L) | 1.56 ± 0.22 | 1.57 ± 0.20 | 1.56 ± 0.21 | 0.034 | 0.967 |
| FEV1/FVC(%) | 48.15 ± 6.21 | 48.24 ± 6.05 | 48.20 ± 6.01 | 0.004 | 0.996 |
| DLCO[ml/(min·mm Hg)] | 15.82±1.61 | 15.91±1.54 | 15.96±1.57 | 0.140 | 0.869 |

**Supplementary Table S2Comparison of lung function between two groups of patients on the day before surgery**

| **Parameter** | **High platelet count (n=106)** | **Low platelet count (n=94)** | **T** | **P** |
| --- | --- | --- | --- | --- |
| FEV1(L) | 0. 84 ± 0. 11 | 0. 85 ± 0. 10 | 1.151 | 0.251 |
| FVC(L) | 1. 57 ± 0. 21 | 1. 55 ± 0. 20 | 0.526 | 0.6 |
| FEV1/FVC(%) | 48. 19 ± 6. 07 | 48. 21 ± 6.13 | 0.026 | 0.979 |
| DLCO[ml/(min·mm Hg)] | 15.85±1.58 | 15.99±1.55 | 0.638 | 0.524 |

**Supplementary Table S3 Comparison of blood routine examination indicators on the first day before surgery**

| Parameter | **High platelet count (n=106)** | **Low platelet count (n=94)** | **T** | **P** |
| --- | --- | --- | --- | --- |
| ESR (mm/h) | 35.83±5.36 | 34.76±4.98 | 1.472 | 0.143 |
| Red blood cell (1×106 /μL) | 5.44±1.59 | 5.32±1.67 | 0.527 | 0.599 |
| Neutrophil (1×103 /μL) | 4.32±1.06 | 4.37±1.08 | 0.35 | 0.726 |
| Lymphocyte (1×103 /μL) | 2.03±0.68 | 2.09±0.71 | 0.665 | 0.507 |
| Eosinophil(1×102 /μL) | 0.28±0.03 | 0.28±0.03 | 0.443 | 0.658 |
| Basophil (1×10/μL) | 0.09±0.03 | 0.09±0.03 | 0.402 | 0.688 |
| Hemoglobin (g/L) | 149.41±24.84 | 149.85±25.37 | 0.124 | 0.901 |
| Platelet (1×10^3^ /μL) | 176.82±42.74 | 267.63±43.13 | 14.924 | p < 0.001 |

**Supplementary Table S4. Multivariate logistic regression analysis of factors associated with adverse wound healing**

| Variable | Coefficient | Std Error | Wald | P Value | OR (95% CI) |
| --- | --- | --- | --- | --- | --- |
| Pre-surgery 1d IL-6 | 0.684 | 0.322 | 2.122 | 0.034 | 1.981 (1.071-3.825) |
| Age (years) | 0.018 | 0.021 | 0.856 | 0.392 | 1.018 (0.977-1.061) |
| Sex (Male vs Female) | -0.312 | 0.487 | -0.641 | 0.522 | 0.732 (0.282-1.901) |
| Tumor Stage |  |  |  |  |  |
| - Stage I (reference) | - | - | - | - | 1.000 |
| - Stage II | 0.425 | 0.532 | 0.799 | 0.424 | 1.530 (0.539-4.341) |
| - Stage III | 0.638 | 0.721 | 0.885 | 0.376 | 1.893 (0.461-7.771) |
| Histology (Squamous vs Adeno) | 0.289 | 0.499 | 0.579 | 0.563 | 1.335 (0.502-3.548) |
| Platelet group (Low vs High) | 0.215 | 0.493 | 0.436 | 0.663 | 1.240 (0.472-3.258) |
| Constant | -3.752 | 1.458 | -2.574 | 0.010 | 0.024 |

Model summary: Nagelkerke R² = 0.089; Hosmer-Lemeshow test p = 0.742
