# Supplementary figures and images for: Impact of perioperative platelet counts and IL-6 on wound healing outcomes after thoracoscopic lung cancer surgery
Source: Ann Med. 2025 Oct 31;57(1):2569993. doi: 10.1080/07853890.2025.2569993 (PMC12581744; doi:10.1080/07853890.2025.2569993)

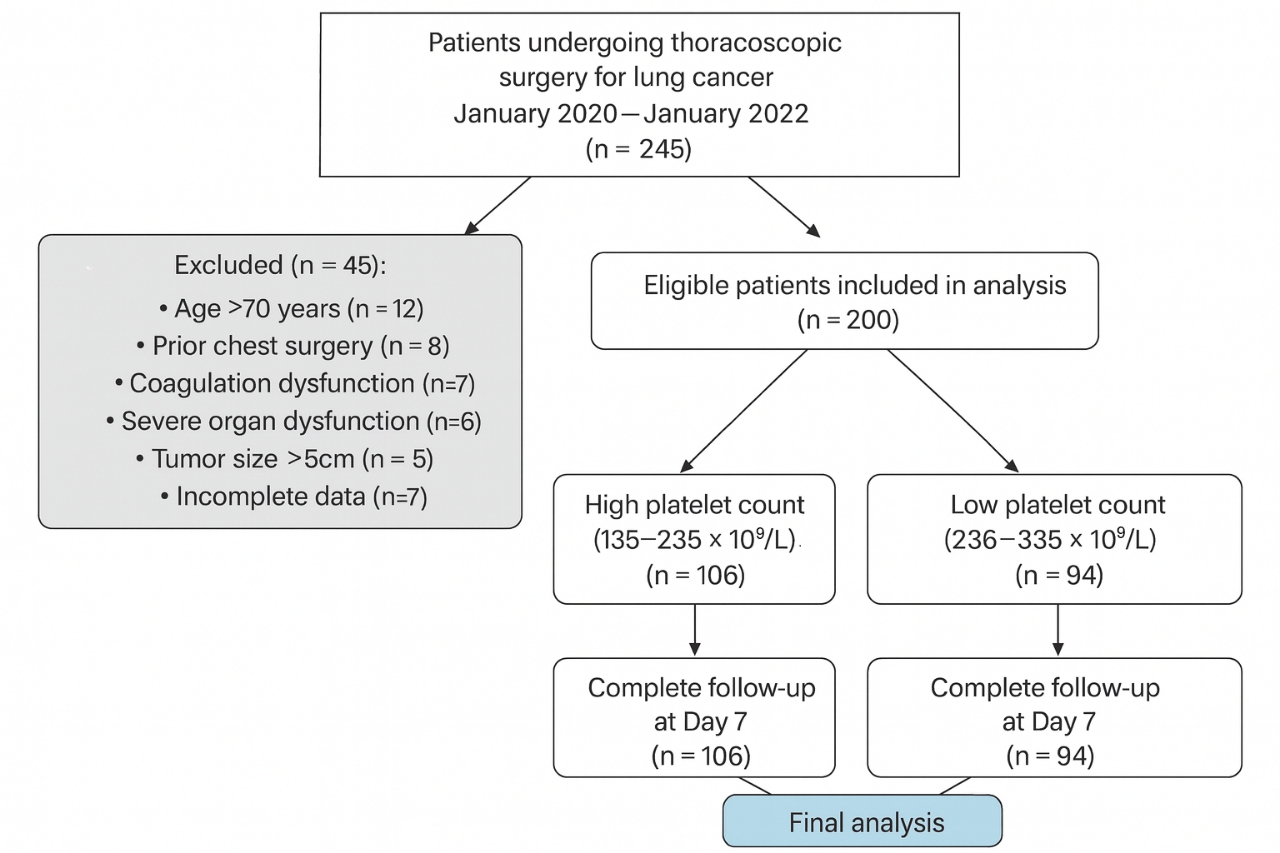

Supplement: Supplemental Material [file IANN_A_2569993_SM1587.jpg]
